# Non-articular sources of groin pain in the athlete

**DOI:** 10.1093/jhps/hnv064

**Published:** 2015-11-09

**Authors:** Ernest Schilders

**Affiliations:** Fortius Clinic,17 Fitzhardinge Street,W1H 6EQ ,London, UK; Leeds Beckett University Headingly Campus, School of Sport, LS6 3QS, Leeds, UK

Groin problems in sports can be multifactorial and often a team approach between orthopaedic, general surgeons is required for both diagnosis and treatment. Both clinical disciplines can be the first point of contact for athletes looking for medical advice.

Good communication between the members of a medical team looking after an athlete is essential and is not helped by the use of confusing terminology.

In 2014, a consensus meeting on Sportsman’s groin was held by the British Hernia Society and it was agreed to use inguinal disruption as the preferred nomenclature [1]. The DOHA group [2] more recently brought together a number of international experts on groin and hip problems in sports with the aim to improve the current taxonomy of groin and hip injuries in sports. It was agreed that terminology should correspond to the anatomical area of the conditions. To describe groin pain, the group, which consisted of orthopaedic surgeons, general surgeons, radiologists, sports physicians and physiotherapist, agreed to use adductor-related groin pain, inguinal-related groin pain, psoas-related groin pain and pubic-related pain.

There is increasing awareness that adductor-related groin pain can coexist with inguinal-related groin pain and conditions such as femoro acetabular impingement. Although these conditions can coexist, it is not clear yet how and if they interact [3]. In patients with long standing adductor-related groin pain, a high prevalence of radiological signs of FAI has been found [4]. It has been suggested that femoroacetabular impingement can initiate adductor-related groin pain and/or inguinal disruption. The mechanism suggested is increased rotation at the symphysis when the hip is brought into an impingement position [5]. It can be a challenge for the treating clinician to decide what to treat and in which order if not simultaneously [6].

In this symposium, three topics will be discussed: inguinal-related groin pain, subspine and rectus femoris impingement and, finally, the overlap between femoroacetabular impingement, the sports hernia and adductor pain.

Inguinal disruption, also called sportman’s hernia, is a common condition in athletes involved in sports such as football, soccer and rugby. The anatomical findings will be discussed, the typical symptoms with which the athlete presents and treatment options.

Other causes of extra-articular hip pain will be discussed such as subspine impingement [7, 8] as well as proximal rectus femoris injuries. Adolescent rectus femoris apophyseal avulsion can predispose the athlete for subspine impingement [9]. The different types of subspinous impingement will be discussed and the intra-articular pathology observed with this condition. Rectus femoris injuries are common and can be complicated with spur/enthesophytes, which can be a cause of extra-articular impingement.

The third paper looks into the hypothesis that femoroacetabular impingement might be the trigger for an inguinal disruption. In addition, the authors report on their management experience of coexisting FAI and inguinal disruption. The team comprised a general surgeon, orthopaedic surgeon and physical therapist.

More detailed knowledge about conditions that coexist with femoroacetabular impingement will help the hip arthroscopist, who deals with athletes, to conduct effective communication with other members of the team. This will significantly improve the athletes’ management and facilitate a faster and more consistent return to sports.

## CONFLICT OF INTEREST STATEMENT

Share holder Fortius clinic. Smith and nephew endoscopy consultant.
